# Psychometric properties of the learning perception questionnaire in Mexican’s students

**DOI:** 10.1038/s41598-022-25912-w

**Published:** 2022-12-29

**Authors:** Raúl Baños, Juan Pablo Machado-Parra, Emilio Arrayales-Millán, Antonio Baena-Extremera

**Affiliations:** 1 Facultad de Deportes, Universidad Autónoma de Baja California, Mexicali, Mexico; 2 Facultad de Ciencias de La Educación, Universidad de Granada, Granada, Spain

**Keywords:** Human behaviour, Psychology

## Abstract

The aim of this study is to analyze the psychometric properties of the learning perception questionnaire (CPA) presented in this research. It was administered to a total of 1496 students in Baja California and Nuevo León, of the total sample, 748 were girls (Mage = 14.0, SD = 0.3), and 748 boys (Age = 14.1, SD = 0.3). The analyses support the hypothesized theoretical model of origin, presenting an acceptable internal consistency and temporal stability. The model fit data was excellent; furthermore, the examined model meets the convergent validity requirements. External validity was explored by examining the predictive relationship of the scale studied with Satisfaction with School. The CPA has a strong predictive relationship with student satisfaction/fun in class, while it is negative with boredom. Thus, the higher the perception of learning, the less likely that students will be bored in class. It is concluded, therefore, that the CPA scale is a proven instrument and that it serves to assess the perception of key learning by secondary school students.

## Introduction

The teaching–learning process is a key issue that all governments and education systems around the world strive to improve. This is why several assessment programs have been designed to examine the students’ learning from different standpoints, for example: the knowledge acquired by the students, the teacher’s competencies, the academic context, among others. Some of these programs have been applied in the Mexican education system, such as the Programme for International Student Assessment (PISA), the Teaching and Learning International Survey (TALIS) or the one proposed by the Secretariat of Public Education (SEP) of Mexico: the National Plan for Learnings Assessment (PLANEA, Plan Nacional para la Evaluación de los Aprendizajes).

The PISA Report is one of the assessments made by the Organization for Economic Co-operation and Development (OCDE) in all its member nations. According to the last OCDE report^1^, Mexican teenagers performed poorly in this program, scoring 420 points in reading comprehension, 409 points in mathematics and 419 points in science, whereas the average of the OCDE countries was 487, 489 and 489 points respectively. On the other hand, international teacher evaluations are also conducted through the Teaching and Learning International Survey (TALIS), which assesses the most efficient competencies for teaching. The main results obtained from this evaluation reveal that teachers in Mexico work in more challenging environments, feel less qualified to do their job and have less academic training and greater participation in professional development^2^. Experts from the Secretariat of Public Education of Mexico (SEP) apply the National Plan for Learnings Assessment (PLANEA) nationwide, whose 2017 report showed that average scores decreased compared to the 2015 baseline; in terms of language and communication, merely 47% of students are at the minimum essential threshold, whereas results in mathematics are even more devastating, with two thirds of students not acquiring the expected learnings^3^. As observed, the various assessments have revealed worrying data in terms of the learnings of Mexican teenagers.

Under such concerning circumstances, the education experts from Mexico’s SEP along with the Secretariat of the Interior approved Decree DOF-30/09/2019 in the Official Journal of the Federation^4^, which sets forth the abrogation of the General Law of Educational Physical Infrastructure, the issuance of the General Law of Education and the creation of the “New Mexican School” (NEM). This educational reform is an evolution from a strategy based on competency to an approach of key learnings expected in the students. Key learnings are sets of fundamental values, knowledges, practices, abilities and attitudes specifically shaped at school that contribute significantly to the comprehensive development of the student^3^, and which, if dismissed, would cause shortcomings hard to offset in crucial aspects of their lives^3^.

In this way, society demands that these learnings be connected to each other so that the adolescents acquires the skills that allow them to solve everyday situations and become competent citizens^5^. The inclusion of these key learnings in the educational framework facilitates the transfer of learning to personal and social development^6^. Along these lines, Blázquez^7^ states that all learning should be applied in a wide range of contexts, with results of high personal and social value, allowing complex demands to be successfully overcome. To do this, the learning of adolescents must be transversal and seek to respond to cognitive, affective and behavioral variables in each school subject^6^.

The NEM considers learnings assessed both in the PISA Report and by PLANEA^3^. In particular, the PISA Report assesses three big fields: reading comprehension, mathematics and science; PLANEA assesses language and communication, whereas the NEM establishes the following as the profile for secondary school graduates: language and communication, mathematical thinking, exploration and understanding of the natural and social world, critical thought and problem solving, socioemotional skills and life project, collaboration and teamwork, coexistence and civility, artistic expression and appreciation, body and health care, environmental protection, and digital abilities.

Scientific literature has revealed that, in recent years, teaching, learning and evaluation processes have been analyzed from the standpoint of teachers, management teams and self-diagnostic tests from government authorities^8^. Teaching programs both at a national^9–11^ and at an international level^12,13^ have also been examined. However, studies on the teenagers’ learning self-perception are scarce; therefore, it is interesting to know what students think about their own learnings^14^, and whether school learnings are preparing them to solve problems or face unexpected situations^15^.

In terms of learning evaluation, the analysis of literature yields a limited number of studies, such as those conducted by Hill et al.^16,17^, who validated a questionnaire with Medicine university students that assessed the perception of learnings acquired for the development of their medical duties. On the other hand, Meroño et al.^8^, designed an instrument that measured the perception of elementary school students regarding the acquisition of basic competencies in a Spanish sample. In addition, Moreno-Murcia et al.^6^ validated a scale with Spanish teenagers that examined the perception in terms of the acquisition of basic competencies at a secondary school level. Moreover, recent studies have focused on the students’ self-assessment of competencies^18–20^. Overall, the international scientific literature is demonstrating the importance of taking students into account for the development of all education-related changes^21^, but for that end, it is needed to devise valid and reliable scales that assess the learning perception in the students.

In this line, the CPA scale validated by Moreno-Murcia et al.^6^, has been a scale used on different occasions in other research studies^22,23^, so we consider it an interesting instrument to apply in the Mexican context. In addition, the CPA has been positively related to the satisfaction of basic psychological needs, intrinsic motivation and the interpersonal style of the teacher^6,22,23^, variables that different studies have highlighted in a positive way, to increase the satisfaction with the school of the student^22–24^. For this reason, we consider it interesting to analyze the relationship between the CPA and academic satisfaction.

Notwithstanding the aforementioned studies, measurement instruments that assess the learning perception of secondary school students are scarce, particularly in the Mexican context^25^. For this reason, two different studies have been proposed, given that in order to carry out this research it was necessary to adapt and validate an internationally contrasted instrument which measures the student's perception of their own learning and the relationship of the said scale to other scales and variables. The purpose of this study is to analyze the psychometric properties of the Learning Perception Questionnaire (LPQ) of Moreno-Murcia et al.^6^ adapted to the Mexican context. As a hypothesis, the CPA is expected to obtain acceptable validity and reliability for its application in secondary schools in the Mexican context. The objective of Study 2 is to analyze the factorial invariance of CPA and whether it predicts satisfaction with school. As a hypothesis, the CPA is expected to be invariant and to positively predict satisfaction with school.

## Study-1

### Method

#### Design

The design of this research was observational, descriptive, cross-sectional and non-randomized^26^.

#### Participants

The sample design was probabilistic by centers, stratified, multistage and by proportional affixation, and was comprised of third-grade secondary school students from the State of Baja California (Mexico). Participating secondary schools were randomly selected using a random number table. The total number of third-grade secondary school students in the State of Baja California was 13,176 girls and 13,627 boys. The representative sample indicated was calculated according to sex for a finite population with a confidence level of 95% and a margin of error of + 5%, consisting of 374 girls (Mage = 14.10; SD = 0.37) and 374 boys (Mage = 14.11; SD = 0.35). The inclusion criteria to participate in this research were: (i) being a student in the third year of Secondary Education. The exclusion criteria were: (i) not having consented to the use of the data in the research and/or (ii) not having completely filled out the data collection form.

#### Instruments

Learning Perception Questionnaire (CPA). To measure the learning perception, we use the questionnaire of Moreno-Murcia et al.^6^, from which it has been designed (intended to be validated) with a single dimension composed of 9 items that measures the perception of expected learnings in different fields, such as: Spanish language, mathematics, scientific-technological, self-learning, initiative and entrepreneurship, cultural expression and awareness, at a social and civic level. The scale was preceded by the phrase “What my secondary school teachers are teaching me allows me to…” (“Lo que me están enseñando mis maestros en la secundaria me permite ser capaz de…”). One sample item was: “Be an autonomous person with own initiative to face challenges and make decisions in my life” (“Ser una persona autónoma y con iniciativa personal para enfrentarme a los retos y toma de decisiones de mi vida”). Answers were compiled using a Likert scale ranging from 1 (completely disagree) to 7 (completely agree). The alpha value found in the study by Moreno-Murcia et al.^6^ was 0.88. Fitness indices found for the confirmatory factor analysis were as follows: χ^2^/gl = 4.67; NFI = 0.91; CFI = 0.93; IFI = 0.93; TLI = 0.91; RMSEA = 0.08 and SRMR = 0.04.

#### Expert validation of the CPA scale

The qualitative analysis of the scale items (content validity) was made based on the assessment of six experts on the Mexican education system at the secondary school level, on educational research methodology and on the university context linked to education. The experts were required to make a value judgment about each of the items in terms of their univocality (yes or no) to examine whether the phrasing of each of the items was unambiguous; appropriateness (yes or no), to analyze whether the questions had a logical correlation with the research object; and importance in a scale of (1 and 2 = completely disagree; 3, 4 and 5 = somewhat agree; 6 and 7 = completely agree) to analyze the fit level of the question in connection to the factor. The SPSS Statistics V.25 statistical software was used for statistical validation, measuring the global agreement from the six experts in terms of univocality, appropriateness and importance through the Intraclass Correlation Coefficient (ICC) based on a mixed-effects model, assuming a definition of absolute agreement. The factor’s Cronbach’s alpha was later analyzed as well. In addition, the agreement dispersion of judges was measured by analyzing the interquartile range, in a manner that, if the difference between quartile Q3 and quartile Q1 was equal to 0 or 1, the item was accepted; if between 1 and 2, the item was revised; and if greater than 2, the item was removed. Values obtained for the interquartile range were mostly 0 or 1.

#### Procedure

In order to carry out this research, a research project called “Programme for International Student Assessment: relationship between school performance in secondary school students and psychological, family and physical activity variables” was first presented to, and later approved and subsidized by the Secretariat of Public Education of Mexico (identification number: 431/569/E). The study was approved by the Ethics committee/Institutional Review Board of Autonomous University of Baja California. Then, authorization was requested from secondary school principals, providing the parents/guardians involved with information for consent detailing the purpose and intentionality of the study. Following their approval, the data collection procedure began by informing the participants of the study’s purpose, that participation was anonymous and voluntary, and that their answers were to remain confidential, reminding them that there were no right or wrong answers, and asking them to answer with complete honesty. All questionnaires were filled inside the classroom in the presence of the lead researcher in case of doubts during the procedure, which lasted 15–20 min.

#### Statistical analysis

To learn the instrument’s reliability, a Test–Retest was performed, which involves applying the questionnaire again to a same sample of individuals in two different occasions, among which the attribute remains stable^27^. The Test–Retest was conducted on 47 third-grade students from a secondary school of the Ensenada municipality; subjects filled the survey twice, with an interval of one week and under the same conditions. Cronbach’s alpha was subsequently used to assess the internal consistency. Next, descriptive, discrimination, normality, linearity and multicollinearity analyses for the scores were carried out prior to analyzing the measurement model and factorial invariance. In particular, the Structural Equation Modeling^28^ was applied through the Confirmatory Factor Analysis (CFA) for the dimensional and internal structure analysis. The maximum likelihood method was used to obtain a better fit of the standard error of model parameters. Among the absolute ones: the *p*-value, associated with the Chi-square statistic (χ^2^), the ratio between and degrees of freedom (gl; χ^2^/gl) and GFI (goodness-of-fit index). Among the relative indexes: NFI (normalized fit index) and CFI (comparative fit index). Also, RMSEA (root mean square error of approximation) was used as incremental indexes^29^. In the case of χ^2^/gl model fit values < 5.0 were considered acceptable, and ratios < 2.0 considered to be indicators of excellent model fit^30^. Values of CFI and NFI > 0.95, RMSEA < 0.06, were considered to be indices of excellent model goodness-of-fit^31,32^. Values of CFI and NFI > 0.90 and RMSEA < 0.08 were considered to be acceptable indices of model goodness-of-fit^31,33^. The analyses referred to were carried out using the statistical software IBM SPSS version 25 and AMOS version 24.

#### Ethical statement

Our research was based on the ethical standards in the WMA Declaration in Helsinki and was approved by the research ethics committee the Secretariat of Public Education of Mexico (identification number: 431/569/E). We informed all participants of the study before the test. The research was conducted after the consent of the participants. In addition, our data were anonymized to ensure the privacy of all participants.

#### Informed consent

Informed consent was obtained from all individual study participants and/or their legal guardians.

### Results

#### Descriptive analysis of the items

The procedure used to analyze each of the CPA scale items followed the analysis guidelines set by Carretero-Dios & Pérez^34^. Said analysis is needed to study the convenience of maintaining each item within each of the theoretical dimensions to which it belongs, according to the original scale^6^. For the study of the items, we analyzed the internal consistency of the extended scale after removing one item, as well as the requirements set out by Nunnally & Bernstein^35^ to maintain an item within a factor: corrected coefficient of item-total correlation (CCIT-c) ≥ 0.30, standard deviation (SD) > 1.0 and all answer choices used at any given moment. As suggested by Hair et al.^36^ data is considered to be normal if skewness is between − 2 and + 2 and kurtosis is between − 7 and + 7. The items from the CPA (see Table 1) showed mean values ranging from 4.16 (item-2) to 5.90 (item-9). SD were > 1. The internal consistency was appropriate (α = 0.84) and did not improve by removing any item. All CCIT-c presented values ≥ 0.38.

**Table 1:** Descriptive statistics, internal consistency and homogeneity (n = 748).

Items	*M*	*SD*	*CCIT-c*	α sin ítem	*S*	*K*
1. Hablar y escribir en español de forma adecuada/correcta. [Speak and write in Spanish adequately/correctly]	5.54	1.53	.57	.83	−.96	.40
2. Comunicarme en inglés para poder desenvolverme en diferentes contextos. [Communicate in English to be able to function in different contexts]	4.16	1.74	.38	.85	−.02	−.74
3. Realizar operaciones básicas y de razonamiento matemático para resolver problemas en la vida cotidiana y académica. [Perform basic operations and mathematical reasoning to solve problems in everyday and academic life]	5.49	1.53	.53	.84	−1.00	.48
4. Analizar, interpretar y obtener conclusiones personales sobre la salud y los avances científicos y tecnológicos. [Analyze, interpret and draw personal conclusions about health and scientific and technological advances]	5.07	1.53	.58	.83	−.77	.09
5. Utilizar recursos tecnológicos para resolver problemas cotidianos de forma eficiente. [Use technological resources to solve everyday problems efficiently]	5.03	1.63	.57	.83	−.72	−.14
6. Expresar lo que pienso respetando a los demás. [Express what I think while respecting others]	5.83	1.54	.64	.83	−1.43	1.41
7. Conocer las distintas expresiones artísticas y culturales para valorar la diversidad. [Know the different artistic and cultural expressions to value diversity]	5.45	1.46	.56	.83	−.94	.39
8. Utilizar adecuadamente técnicas de estudio así al finalizar mis estudios continuaré aprendiendo de forma independiente y eficaz. [Properly use study techniques so that at the end of my studies I will continue to learn independently and effectively]	5.78	1.45	.65	.83	−1.50	1.93
9. Ser una persona autónoma y con iniciativa para tomar decisiones y enfrentar las adversidades de mi vida. [Being an autonomous person with initiative to make decisions and face adversities in my life]	5.90	1.46	.65	.83	−1.71	2.61

N, sample; M, mean; SD, standard deviation; CCIT-c, corrected coefficient of item-total correlation; S, skewness; K, kurtosis.

#### Temporary Stability

Temporal stability was analyzed using the test–retest method in a sample of 47 students, 23 girls (Mage = 14.02; SD = 0.35) and 24 boys (Mage = 14.07; SD = 0.37). The sample selection was for convenience. The second assessment with the d2 test was performed after the approximate median time interval of two months (M = 1.88; SD = 0.75) from the first assessment. The normality analysis indicated that the CPA scores resulting from the first and second evaluation presented a normal distribution. The result of the Pearson correlation was 0.92 for the CPA (*p* < 0.01).

#### Confirmatory factor analysis

First, a multivariate normality analysis was performed on the CPA scale. The normality test was carried out based on the Mardia´s coefficient from the AMOS v.24. Given the violation of the assumption of multivariate normality (Mardia's coefficient = 30.167; *p* < 0.001), this analysis was performed with the maximum likelihood method and the bootstrapping procedure of 5000 iterations^37^. The polychoric correlation and asymptotic covariance matrices were used as input for the data analysis. A single factor measurement model was hypothesized.

**Figure 1:**
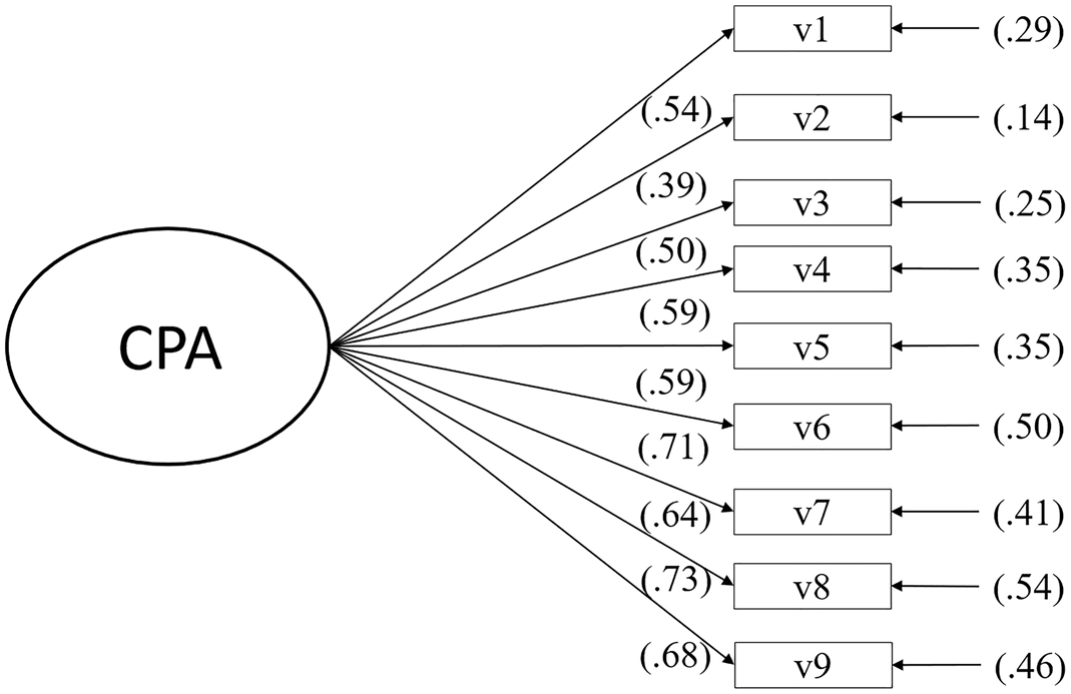
This path diagram shows the factorial loads of each of the items that make up the CPA (Learning Perception Questionnaire) scale.

The tested model exhibits high factorial loads (≥ 0.39). All fitness data were acceptable (χ^2^ = 126.895, gl = 26, *p* = 0.000, χ^2^/gl = 4.881, GFI = 0.961, NFI = 0.935, CFI = 0.948, RMSEA = 0.072). All factor loadings were > 0.40 (see Fig. 1), with the exception of item-2 (0.39).

Regarding the CFA of the scales with ordinal nature of the data correlation matrix, it is also important to present the results of composite reliability. According to Hair, Black, Babin and Anderson^36^, the minimum values for composite reliability should be .70. The tested model had a composite reliability of .84.

## Study-2

### Method

#### Design

The design of this research was observational, descriptive, cross-sectional and non-randomized^26^.

#### Participants

The sample design was probabilistic by centers, stratified, multistage and by proportional affixation, and was comprised of third-grade secondary school students from the State of Nuevo León (Mexico). Participating secondary schools were randomly selected using a random number table. The total number of third-grade secondary school students in the State of Nuevo León was 13,396 girls and 13,831 boys. The representative sample indicated was calculated according to sex for a finite population with a confidence level of 95% and a margin of error of + 5%, consisting of 374 girls (Mage = 13.99; SD = 0.30) and 374 boys (Mage = 14.02; SD = 0.33). The inclusion criteria to participate in this research were: (i) being a student in the third year of Secondary Education. The exclusion criteria were: (i) not having consented to the use of the data in the research and/or (ii) not having completely filled out the data collection form.

#### Instruments

Learning Perception Questionnaire (CPA). The instrument has been described in study 1.

Satisfaction with School: To measure satisfaction with school (SWS), we used the scale adapted to the Mexican context by Baños et al.^38^, from the original by Duda and Nicholls^39^ to identify satisfaction and boredom at school. The instrument consists of eight items that measure the satisfaction/enjoy degree (five items) and the boredom degree (three items). The scale was preceded by the phrase “Tell us how much you disagree or agree with the following statements, in terms of all your school subjects” (“Dinos tu grado de desacuerdo o acuerdo en relación a las siguientes afirmaciones, referidas a todas tus clases en el instituto”). Answers were collected using a scale of polytomous items ranging from 1 (completely disagree) to 5 (completely agree). The internal consistency found in the research by Baños et al.^38^ was: satisfaction/enjoyment: α = 0.66; boredom: α = 0.65, and fit indices found for the confirmatory factor analysis were: χ^2^ = 20.49; gl = 13; *p* = 0.08; χ^2^/gl = 1.57; GFI = 0.99; NFI = 0.94; NNFI = 0.96; CFI = 0.98 and RMSEA = 0.04.

#### Procedure

The procedure was similar to that in study 1.

#### Statistical analysis

In this second study, first, multivariate normality was calculated. Given the violation of the assumption of multivariate normality (Mardia's coefficient = 44.02; *p* < 0.001), this analysis was performed with the maximum likelihood method and the bootstrapping procedure of 5000 iterations^37^. The goodness of fit was evaluated with < 5.0 for the chi-square coefficient and degrees of freedom (χ^2^/df), values > 0.90 for the CFI (Comparative Fit Index), TLI (Tucker–Lewis Index).. Subsequently, measurement factorial invariance analysis was conducted following the advanced methodological approach by Milfont & Fisher of testing four increasingly more constrained models^40^. This is why four progressively more restrictive nested models were considered: (1) Model 1, configural invariance (M1, no restrictions), which served as a benchmark for the other models, (2) Model 2, metric invariance (M2, restriction on factorial loads); Model 3, strong invariance (M3, restriction on factorial loads and intercepts) and Model 4, strict invariance (M4, restriction on factorial loads, intercepts and error variance)^41^. Lastly, a structural regression model was created to study the prediction of satisfaction with each of the school subjects based on the students’ perception on their teachers’ competencies. These analyses were carried out using the statistical software IBM SPSS version 25 and module AMOS version 24.

#### Ethical statement

Our research was based on the ethical standards in the WMA Declaration in Helsinki and was approved by the research ethics committee the Secretariat of Public Education of Mexico (identification number: 431/569/E). We informed all participants of the study before the test. The research was conducted after the consent of the participants. In addition, our data were anonymized to ensure the privacy of all participants.

#### Informed consent

Informed consent was obtained from all individual study participants and/or their legal guardians.

### Results

#### External validity

A regression analysis was performed to test to what extent the CPA (independent variable) predicts the SWS dimensions (dependent variable) (see Table 2). The CPA is proven to show a strong positive prediction correlation with student satisfaction/enjoyment during class, hence the more students perceive to be acquiring key learnings, the more enjoyment students will experience at school. The correlation is negative with boredom, that means, when students do not perceive to be acquiring key learnings, boredom at school is predicted.

**Table 2:** Simple linear regression of the CPA (independent variable) predicts the dimensions of the SWS (dependent variable).

Dimensions	*F*	*ß*	*p*
Satisfaction/enjoy	33,834	.21	.000
Boredom	8481	−.11	.004

*ß* = Standardised beta weights

**p < .01.

#### Factorial invariance according to sex

Invariance according to sex (i.e., 374 girls, 374 boys) of the CPA was tested based on CFA models. The results from the invariance tests are shown on Table 3. The models (configural invariance, metric invariance, strong invariance and strict invariance) provide a proper goodness-of-fit level (CFI > 0.90, TLI > 0.90 and RMSEA < 0.08) and do not exceed the cut off points recommended for comparison of the increasingly restrictive models for RMSEA (Δ < 0.015), CFI (Δ < 0.01) and TLI (Δ < 0.01).

**Table 3:** Goodness of fit indexes of the invariance models.

Modelo	χ^2^	*df*	RMSEA [90% IC]	CFI	TLI	Comparación de modelos	Δχ^2^	Δ *df*	ΔRMSEA	ΔCFI	ΔTLI
M1	200.285	54	.060 [.051–.069]	.934	.912	–	–	–	–	–	–
M2	210.813	62	.057 [.048–.065]	.933	.922	2 versus 1	10.528	8	.003	.001	− .01
M3	234.582	71	.055 [.048–.063]	.926	.925	3 versus 2	23.769	9	.002	.007	− .003
M4	279.981	81	.057 [.050–.065]	.920	.920	4 versus 3	45.399	10	− .002	.006	.005

χ^2^, Chi-square; df, degrees of freedom; RMSEA, root mean square error of approximation.; CFI, confirmatory fit index; TLI, Tucker–Lewis index; *p < .01; Model 1, configural invariance (M1, no restrictions), Model 2, metric invariance (M2, restriction on factorial loads); Model 3, strong invariance (M3, restriction on factorial loads and intercepts); Model 4, strict invariance (M4, restriction on factorial loads, intercepts and error variance).

## Discussion

The proposal for this research came from the need of acquiring and bringing new knowledge to the secondary education environment in Mexico, with the students’ perception on their learnings as the main focus. It is worth mentioning that the drafting of the items has taken into account the contents assessed in the PISA Report, the PLANEA evaluation and the NEM, which establishes what are the attributes that secondary school graduates in Mexico should possess. The objectives of this study were two: (a) to analyze the psychometric properties of the Learning Perception Questionnaire (LPQ) of Moreno-Murcia et al.^6^ adapted to the Mexican context, presented in this research; (b) analyze the factorial invariance of the CPA and whether it predicts satisfaction with the school. The two hypotheses have been confirmed, the CPA is a valid and reliable instrument for its application in secondary schools in the Mexican context, and the CPA is an invariant scale that positively predicts satisfaction with the school.

The data analysis revealed the CPA internal consistency to be adequate; this value would only increase slightly by having item-2 removed, but according to Nunnally & Bernstein^35^, this factor is not considered relevant enough to justify the removal of the item. It is worth mentioning that all other indicators suggest keeping all items of the scale, such as CCIT-c, deemed acceptable for Nunnally & Bernstein^35^. Furthermore, the results obtained for factorial loads are high, which indicate belonging of each of the analyzed items to the factor, as well as the individual reliability values, all of them above the minimum required threshold.

The fitness data obtained in this study are considered excellent according to Hooper et al.^31^. Other studies conducted with instruments that also assessed the perception of learning in students did not obtain a construct validity fit as adequate as the one obtained by this research; for example, the instrument designed by Moreno-Murcia et al.^6^ with Spanish secondary school students, the instrument intended for elementary school students also in the Spanish context by Meroño et al.^8^; and lastly, the learning perception instrument designed for Chinese university students by Yu et al.^17^. It is important to mention that the referred studies did not mention all fitness parameters that were mentioned in this research. The validity results obtained in this research prove an acceptable goodness-of-fit index in measuring the learning perception of secondary school students in Mexico, and even with a higher level of validity when compared to the design of other questionnaires that measure the same variable at an international level^6,8,17^, making a great contribution to the Mexican Education System.

In terms of the factorial invariance analysis according to sex, factorial invariance of the CPA is confirmed in factorial loads, intercepts and error variance, therefore the CPA can be considered a valid instrument to conduct mean comparison studies according to the sex variable. What is innovative in this study is the addition of the factorial invariance analysis according to sex, not conducted in any of the instruments examined as per the theoretical framework that also measured perception of learning from the students^6,8,17^. Nonetheless, it is recommended to conduct further studies using this questionnaire, analyzing the factorial invariance at different school levels (such as elementary and higher education) across different states of Mexico.

The SWS was used for the external validity analysis due to the relationship between both variables and the potential practical applications arising from said relationship^25,42^. Results show that the CPA positively predicts satisfaction and enjoyment at school, and negatively experiences related to boredom at school. However, because no references have been found to other studies that analyze these relationships, these results must be interpreted with caution. An explanation for this relationship between the student’s learning perception and satisfaction with school might stem from the competencies acquired by teachers^43^, the combination of strong relationships among the student’s family and high levels of school satisfaction, understood that these serve as a stimulus for the academic success of teenagers^44,45^. Therefore, the school policies and practices that improve the teachers’ competencies, school satisfaction and family relationships may increase the learning perception from the students.

## Conclusion

As a conclusion, it can be attested that the CPA scale meets the necessary requirements for its validation, settling as a reliable and solid unidimensional scale. The obtained results endorse the use of CPA as they are coherent with the few existent research efforts both in the university academic environment and in the elementary education levels in Mexico, and with the logical relations of other scales, such as SWS.

The validation of this instrument in the Mexican context will facilitate the assessment of learning perceptions from secondary school students. In this manner, the opinion of students could be considered for future amendments to education laws and models by being aware of what students think about their own learnings^14^ and whether what they learn in the school environment is training them to solve problems or face unexpected situations^15^. In addition, the validation of this scale to the Mexican context will allow the teachers to know in the first instance, whether among the key learnings they are imparting in the classroom the students perceive the ones useful to solve daily problems that they find in their lives.

Lastly, it would be interesting to see future research efforts examine the relationship between the student’s learning self-perception with other variables, such as teachers’ competencies, satisfaction with the various subjects, academic performance or the teenagers’ emotional intelligence. It would also be interesting to know the validity and reliability of the CPA when applied to other education levels.

## Limitations and strengths

This research faces a series of limitations that should be mentioned. The CPA scale can be applied to the secondary school level regardless of the subject’s gender, however, it cannot be claimed that said scale can be used neither at different school levels nor in other states of Mexico, given that the study population was not representative of the entire country. Because of this, the results from this research cannot be generalized. Notwithstanding the above, a strength to underscore is the sample design, probabilistic and random by centers, stratified, multistage and by proportional affixation. In this manner, the study results can be generalized for the State of Nuevo León and Baja California, Mexico. In addition, another important strength is the addressed topic, as it can greatly contribute to generate solutions in terms of the main issues related to the learnings of teenagers at school.

## Data Availability

The data sets generated and/or analyzed during the current study are not publicly available due to data protection law and because the data in this work belongs to a larger project that has not yet been completed, but are available from the corresponding author at reasonable request.
